# The Twentieth Annual Meeting of the Pennsylvania State Society

**Published:** 1888-08

**Authors:** 


### THE 20TH ANNUAL MEETING OF THE PENNSYLVANIA STATE SOCIETY.

The Pennsylvania State Dental Society held its annual meeting at Philadelphia, June 5th, 6th and 7th. Although occurring at the busiest season for city dentists, yet the attendance was very good indeed, especially at the afternoon sessions. Owing to the enforcement of the rule restricting the privileges of the floor to members who had paid all arrearages, invited guests and non-residents, the membership was considerably increased. An amusing incident occurred when the editor of one of the dental journals published in the city, not knowing of the arbitrary resolution, attempted to make some remarks. He was called to order by the President, who said that the gentleman would have to pay his annual dues before he could proceed. The doctor marched up bravely and paid his fees, and as his speech was short, it cost him about three dollars per minute. It remains to be seen whether such resolutions will not, in the long run, react upon the society. The success of all public bodies depends upon their liberality and public spirit. A resolution that deprives an association from the benefits of outside instruction by allowing none but members to address it smacks of narrow-mindedness. Under the restrictions adopted the Association shut itself out from deriving any benefit from the other professions, no matter how desirable it might be to have such addresses. Too much time was given to the consideration of constitution and by-laws, and doubtless served to keep many away from the meetings. The address of the President, which appears elsewhere in this number, should be read. Dr. Fundenberg has grasped the vital question of the day, and his remarks should receive thoughtful consideration by the profession. Prof. Truman sounded a warning note in regard to the use of gold, and the decadence of manipulating ability. His paper brought out plainly the methods practiced in the three dental colleges in Philadelphia. Dr. Register, however, proved the dark horse of the convention, and won praise for his well-timed words regarding the necessity of a knowledge of the histological structure of the teeth. No one suspected him of having a presidental bee in his bonnet, and he says that he had not thought of it, but nevertheless, the Association elected him president for the ensuing year. Dr. Faught's paper contains much food for thought, and, as Dr. Truman remarked, if the figures were not before us, it would hardly be possible to believe that the writers in the profession were so few. Dr. Kirk's paper was a classical presentation of the subject of implantation, based upon personal experience in over thirty cases. Dr. Pierce brought out the subject of impaction of the third molars, and the causes which led thereto, prefacing his remarks by a brief summary of the genesis and eruption of the permanent teeth. Dr. Magill's and our paper completed the list.

Perhaps the most enjoyable feature of the session was the excursion down the river to Fort Delaware and return, on the steamer John Walton. Two hundred invitations, including the wives and lady friends of the delegates, had been issued. The fair sex responded to the invitation, and appeared greatly to enjoy the afternoon's outing. Dr. Allen and son and Dr. Parr, of New York, were guests of the Association. A string band and the Harmonia Glee Club made sweet music, and lemonade without any stick was dispensed with a prodigal hand. An elegant supper was served on the lower deck, and was fully enjoyed. After all had finished, the audience adjourned to the spacious saloon, and listened to a clear and entertaining explanation of the points of interest that so thickly stud the banks of the historic Delaware. Dr. Kingsbury fairly outdid himself as he warmed up to the narration of the exciting events of our national history, and demonstrated that a good after-dinner speech may be made without the flow of champagne. On the whole, the excursion was one of the pleasantest in which we remember of ever having partaken. It was the generous gift of the profession of Philadelphia to the visiting members from other portions of the State.

